# Women with Hemophilia: Case Series of Reproductive Choices and Review of Literature

**DOI:** 10.1055/s-0041-1730036

**Published:** 2021-06-01

**Authors:** Shadan Lalezari, Assaf A. Barg, Rima Dardik, Jacob Luboshitz, Dalia Bashari, Einat Avishai, Gili Kenet

**Affiliations:** 1 National Hemophilia Center, Sheba Medical Center, Tel Hashomer, Israel; 2 Sackler Faculty of Medicine, Tel Aviv University, Tel Aviv, Israel; 3 Amalia Biron Research Institute of Thrombosis and Hemostasis, Sheba Medical Center, Tel Hashomer, Israel

**Keywords:** hemophilia, carriers, women, pregnancy, childbirth, reproduction

## Abstract

**Aim** Very little is known regarding reproductive choices, pregnancy, and delivery of women with moderate to severe hemophilia. Our aim was to describe our experience with three hemophiliac women and their journey to achieve motherhood.

**Methods** Medical charts of women with moderate to severe hemophilia A treated at our center were evaluated. Data regarding choices of conception, pregnancy course, mode of delivery, and pregnancy outcomes were obtained.

**Results** Three women are presented. Whereas patient 1 chose to adopt her first child and later had twins through egg donations and a surrogate mother, patient 2 underwent spontaneous pregnancy and delivered via cesarean section. Patient 3 preferred in vitro fertilization and preimplantation genetic diagnosis to avoid hemophilia and hemophilia carriership in her offspring.

**Conclusion** The appropriate means to achieve parenthood for women with moderate to severe hemophilia should be individualized and requires support of a comprehensive multidisciplinary team.

## Introduction

Hemophilia A (HA) occurs in 1:5,000 live male births. As HA is a recessive X-linked disorder, it is historically considered a disease affecting males. Females may be heterozygous for the mutation and referred to as hemophilia carriers. The carriers are expected to have plasma concentrations of factor VIII (FVIII) approximately half the concentration of healthy individuals, providing adequate hemostasis. 1 However, a wide range of clotting factor levels is seen in carriers and those with FVIII activity <0.6 IU/mL may be symptomatic with various bleeding manifestations, 1 despite a level of 0.4 IU/mL being the upper limit defining hemophilia. 2 The bleeding symptoms may include cutaneous bruising and hematomas, heavy menstrual bleeding, hemorrhagic ovarian cysts, hemarthrosis, a higher risk of prolonged bleeding after operations, tooth extractions, and increased risk of postpartum hemorrhage (PPH). 1 3 4 5 This risk is inversely correlated with plasma FVIII levels. 1 4

There are different estimations of hemophilia prevalence in women. Some estimate that 1:100,000 women is a symptomatic HA carrier. 6 However, severe HA (FVIII < 0.01 IU/mL), a condition that places a woman at risk of severe bleeding complications, is an extremely rare event. This phenotype may stem from extreme lyonization (inactivation) of the unaffected X chromosome, partial or complete monosomy of the X chromosome (Turner syndrome) and compound heterozygosity or homozygosity in female progeny of hemophilia carriers and affected hemophiliac male. There are cases of other rare occurrences of hemophilia in females such as phenotypic females with testicular feminization (46XY), combined factor V and FVIII deficiency and acquired hemophilia. 7

As pregnancy and delivery among women with hemophilia are extremely rare, we examined the reproductive choices of female hemophilia patients at our hemophilia treatment center (HTC). Additionally a literature search and review has been conducted and is presented here.

## Methods and Case Reports

The Israel National Hemophilia Treatment Center follows and treats approximately 600 HA patients. Among these are five females who were diagnosed at an early age with moderate to severe HA. Genotyping has shown that one patient is 45 × 0, Turner syndrome. The other four patients are hemophiliacs due to extreme lyonization of their unaffected X chromosome. Three of these women chose to have children and we hereby present their different ways to accomplish motherhood. All three women are followed and treated at our center and received a comprehensive multidisciplinary care by genetic counselors, obstetricians, hematologists, and the psychosocial team before and during pregnancy and after childbirth.

We reviewed the medical records of our three female HA patients who are mothers. Clinical history, family history, FVIII activity, and genetic analyses were collected. Documentation was obtained regarding reproductive decisions, pregnancy follow-up, delivery, postpartum course, and offspring outcomes. The retrospective study of patients' medical files from our computerized database was approved by our institutional review board in concordance with the Declaration of Helsinki.

### Patient #1

Patient was born to a father with severe HA. She was diagnosed with moderate HA (FVIII level: 4%) at the age of 2 years, due to cutaneous hematomas and a joint bleed. The genetic analysis revealed intron 22 inversion with extreme lyonization of the other X-chromosome. At the age of 6 years, she was diagnosed with a brain tumor and underwent craniotomy for tumor removal, followed by chemotherapy and radiotherapy.

In the 1970s, as a young hemophilia patient, she was treated with cryoprecipitate and plasma-derived FVIII and was infected with human immunodeficiency virus (HIV) and hepatitis C virus (HCV). Her viral load has been minimal since the introduction of antiretroviral therapy with a normal CD4/CD8 ratio. She was later treated for HCV and achieved a complete viral response. She continued with mainly on demand treatment for her rare hemarthroses, with prophylaxis before surgical and dental procedures.

At the age of 28, the patient became pregnant following in vitro fertilization (IVF) with preimplantation genetic diagnosis (PGD). However, pregnancy was terminated due to marked elevation of liver enzymes and the couple was obliged to refrain from further attempts. At the age of 30 they adopted a healthy infant. A few years later, they had twins from IVF utilizing egg donations with the husband's sperm. This was materialized through a surrogate mother.

### Patient #2

This patient with hypothyroidism and sporadic moderate HA (FVIII level: 3%) is known to our hemophilia center since early infancy due to recurrent hemarthrosis. She was later found to be a carrier of intron 22 inversion with extreme inactivation of the other X chromosome. As family history was negative for hemophilia and the mutation was not found in her father's peripheral blood DNA, it was postulated that the mutation was either a de novo germ cell mutation or germ cell or somatic mosaicism. 7 8 9 Paternity was confirmed through genetic linkage analysis.

This patient suffered from recurrent hemorrhagic ovarian cysts whenever she stopped oral contraceptives. Menstruation was controlled with oral contraceptives and occasional FVIII replacement in the beginning of the menses.

Once she was married, the options regarding conception were discussed, prenatal diagnosis or IVF with PGD were offered. Yet she conceived spontaneously at the age of 24 years, refusing any intervention or diagnostic evaluation of the hemophilia status of the male embryo.

She received intravenous (IV) prophylactic recombinant FVIII 2,000 IU (35 IU/kg) every other day since early pregnancy. Her plasma FVIII trough levels were in the range of 10 to 20 IU during pregnancy. She was followed at our hemophilia center and at high-risk pregnancy unit for prenatal care.

Toward the end of her pregnancy, delivery options were discussed to protect both the mother and the potentially hemophiliac male newborn. Vaginal delivery was planned, with the intent to switch to a cesarean section early in the course of any complication, avoiding instrumental delivery. However, a semi-elective cesarean section was performed at 38 weeks' gestation under general anesthesia. She received an IV FVIII bolus of 2,000 units, yielding a FVIII level of 95 IU at commencement of surgery. In addition, she received IV 1,000 mg tranexamic acid and local fibrin sealants were applied on the surgery surfaces. Postdelivery FVIII and tranexamic acid were continued for 2 weeks and then regular prophylaxis was resumed. The baby was diagnosed with severe HA. Currently both mother and child are being followed at our center and both receive FVIII prophylaxis.

### Patient #3

This is a 35-year-old patient with severe familial HA (her father and her paternal uncle were both treated at our center with an IVS 5 + 2 T > G splice site mutation).

She was diagnosed with severe HA at an early age due to hemarthrosis. Most of her life she received on-demand treatment for bleeds, mainly joint bleeds.

Prior to pregnancy she underwent a thorough genetic investigation and counseling and chose to proceed with IVF with PGD with a female embryo not carrying the *FVIII* gene mutation.

She started prophylaxis with IV FVIII 2,000 units (35 units/kg) twice weekly from early pregnancy, with FVIII trough levels in the range of 3 to 7 IU. Her pregnancy was uneventful until the 38th week when she was hospitalized with preeclampsia. Following administration of 1,000 mg tranexamic acid and an IV bolus of 2,000 IU FVIII, resulting in a FVIII level of 82 IU, an epidural anesthesia was performed. She underwent emergency cesarean section due to nonreassuring fetal monitoring and meconial water. Intensified prophylaxis was maintained for the first week and tranexamic acid was continued for a total of 2 weeks.

## Discussion

Literature regarding pregnancy and delivery in women with hemophilia is sparse reflecting the rarity of this event.

The patients in the current study were offered genetic counseling prior to conception to enable them to have healthy offsprings. This can be achieved by prenatal diagnosis of spontaneous pregnancies (and considering termination of pregnancy in case of severe hemophiliac fetus) or IVF with PGD.

Patient #1 faced additional challenges due to her HIV and HCV infection. Notably, assisted reproductive technologies are offered for HIV serodiscordant couples with hemophilia. 10 11 Following an unsuccessful pregnancy attempt, they chose to adopt and later had twins from donor oocytes, inseminated by the husband's sperm and a surrogate mother. Thus they materialized their wish to have a family and they put an end to the hemophilia gene in their progeny.

Patient #2 is an orthodox Jew and chose to avoid any interference with natural reproduction. Indeed, often the decisions made by hemophilia carriers are influenced by the families' cultural and religious background. 12 Patient #3 chose to undergo IVF with PGD to ensure a nonhemophiliac offspring. It has been suggested that previous hemophilia experience may affect reproductive choices. 13 Interestingly, other family members of this patient suffered from severe consequences of hemophilia.

In the current study, all three women were hemophiliacs due to extreme lyonization. This is an extremely rare event of marked nonrandom inactivation of the X-chromosome. Extreme lyonization in a carrier of HA may indicate carrier status of several X-linked disorders on the nonhemophiliac X chromosome, including mental retardation and immune disorders. 14 Thus, in cases of women with severe hemophilia due to extreme lyonization, establishing a genetic cause of the nonrandom skewing is of great importance, as the male offspring will either be hemophiliac or at risk of another X-linked disease. The female offsprings will either be carriers of hemophilia or carriers of another X-linked disease. The maternal skewed X chromosome will probably be silenced in the female progeny. However, her sons, carrying the nonhemophilic X chromosome, may be inflicted by a serious disease.

Patient #3 chose a female fetus carrying her extremely skewed X chromosome, assuming that it will be lyonized in the female offspring, as it was in the patient and her mother. Extreme lyonization was confirmed in the female baby after birth. One may argue that carrying a hemophilia gene was a safer decision rather than choosing the X chromosome harboring another unknown disease. This is especially relevant nowadays as hemophilia is a treatable and perhaps a curable disease. 15

Lusher et al reported on four deliveries of four women with moderate to severe HA, one of them had excessive bleeding during and after delivery, necessitating blood transfusions. However, there is no information regarding mode of analgesia, delivery, and peripartum treatment of these women. 16 Table 1 summarizes the latter case reports from the literature and our two patients who became pregnant and delivered. 6 17 18

**Table 1:** Case reports of pregnancy management, labor, and postpartum treatment of women with HA

	Hemophilia severity	IV FVIII Tx during pregnancy	Delivery	Analgesia	IV FVIII Tx—delivery and postpartum	Offspring
Dhar et al 6	Severe	Prophylaxis 30 IU/kg × 2/week	Spontaneous vaginal	Epidural	Bolus 50 IU/kg then IV CI 3 IU/kg/h for 3 days, then regular prophylaxis	Female, not hemophilia carrier
Sharma et al 17 (2 pregnancies of the same woman)	Severe	Prophylaxis 40 IU/kg × 2/wk	Induced vaginal	General anesthesia	25 IU/kg × 3/day 25 IU/kg × 2/day (days 2–5), 25 IU/kg/d (days 6–14), every other day for another 4 weeks	Female, female
Bodrozic et al 18	Severe	On demand for bleeds 20 IU/kg	Planned cesarean section	General anesthesia	50 IU/kg predelivery. Postdelivery: 30 IU/kg × 2/day for 6 days, tranexamic acid (for 6 weeks) and hormonal contraceptives	Female, not hemophilia carrier
PT #2	Moderate	Prophylaxis 35 IU/kg × 3/wk	Semi-elective cesarean	General anesthesia	2,000 IU FVIII × 2 on day of delivery, 2,000 IU × 1/day for a week and every other day for another week, tranexamic acid 1 gr × 3/day for 2 weeks	Male hemophiliac
PT #3	Severe	Prophylaxis 35 IU/kg × 2/wk	Emergency cesarean section	Epidural	2,000 IU × 2 on day 0, then 2,000 IU × 1/day for 3 days, 2,000 IU every other day for a week + tranexamic acid for 2 weeks	Female, not hemophilia carrier

Abbreviations: CI, continuous infusion; FVIII, factor VIII; IU, international units; IV, intravenous; Tx, treatment.

The risk of bleeding in early pregnancy is unknown in carriers of hemophilia. The risk of miscarriage in this population was reported to be 31% (vs. 12–13.5% in the general population), with the risk of antepartum hemorrhage not increased. 19 One should keep in mind that as pregnancy progresses, FVIII levels increase in pregnant women. In hemophilia carriers with borderline FVIII levels, this increase can normalize the FVIII levels. However, in women with moderate to severe HA, this increase is not sufficient to allow for delivery without replacement therapy. 20 We initiated prophylaxis during first trimester and continued FVIII replacement throughout the pregnancies. None of our patients experienced any bleeds during pregnancy in concordance with previous published reports ( Table 1 ).

In the guidelines regarding labor of women with inherited bleeding disorders, 21 regional anesthetic block is not contraindicated provided normal coagulation screen and the relevant factor level above 50 IU/dL. Indeed, one of our patients gave birth under general anesthesia and one with an epidural block. There is a case report on one other woman with hemophilia who gave birth with an epidural block 6 while the other four women gave birth under general anesthesia ( Table 1 ).

The safest method of delivery of fetuses at risk of having a coagulation disorder is still controversial. 22 Davies and Kadir reported that vaginal delivery is associated with an increased risk of intracranial hemorrhage (ICH) as compared with cesarean section. However, a recent PedNet study showed that vaginal delivery and cesarean section carry similar risks for ICH and other major bleeds. 23

When fetal sex or coagulation status is unknown, or in case of an affected male fetus, the use of any invasive monitoring, and instrumental delivery are contraindicated. Among the six deliveries presented in Table 1 , three deliveries were vaginal and three cesarean sections were performed.

Hemophilia carriers are at increased risk of both primary (>500 mL blood loss within the first 24 hours) and secondary (after the first 24 hours) PPH, with an incidence of 19 and 2% respectively as opposed to 5–8% and 0.8% in the general population. 20 Regular follow-up at HTC and high-risk pregnancy units and having a delivery plan prepared in advance reduce the occurrence of peripartum complications. Our patients continued treatment with FVIII aiming at levels above 50 IU/dL in the first 3 days postvaginal delivery and the first 5 days post cesarean section, in accordance with guidelines 21 and then were gradually treated with smaller doses and less frequently until 2 weeks postdelivery when regular prophylaxis was resumed.

The current small cohort of females with moderate to severe HA presents a variety of reproductive issues. Our data support the adequacy of guidelines regarding pregnancy and delivery for the unique group of women with hemophilia. Materializing the women's wish to motherhood can be achieved safely with multidisciplinary care of the women and their newborns.
